# Update on smoking cessation techniques

**Published:** 2010

## Introduction

‘While public attitudes to smoking have changed radically, medical approaches are often outdated and need to be reviewed to resolve tobacco addiction in a strategic and sensitive manner.’ Dr Andrew Pipe of the Heart Institute, University of Ottawa, Canada presented this view in a series of lectures to South African clinicians and physicians at the recent SA Heart Congress.

Reasons for considering renewed clinical efforts to encourage patients to stop smoking include important motivating factors. ‘In the management of patients with post-myocardial infarction, smoking cessation is the most powerful intervention available to the clinician. It reduces mortality by 36%, compared to statin therapy, which reduces mortality by 30% and blood pressure control using β-blockers and other agents, which reduces mortality by 23%.’

Intensive in-hospital smoking cessation programmes1 have been shown to result in a dramatic reduction in hospital re-admissions and all-cause mortality in the 24-month period following the initial event. These effective programmes were conducted for only a 12-week period.^2^ Smoking cessation programmes and the use of modern therapeutic agents are cost effective and result in an annual cost of between $2 000 and $6 000, while treatment of hypertension averages $9 000 to $26 000, and lipid-lowering strategies some $50 000 or more.

A further relevant consideration is that smoking diminishes the benefits of statin therapy. There is a 61% increased risk of cardiovascular events in smokers compared to statin-treated non-smokers.

Mortality from chronic obstructive pulmonary disease (COPD) ranks as the fourth leading cause of death worldwide. The benefits of stopping smoking in these patients include an overall improvement in life expectancy of six to seven years.

‘Tobacco is truly addictive, as the nicotine reaches nicotine receptors in the brain stem very quickly, causing an increase in dopamine in the forebrain, which cements the situational environment in favour of smoking. Our interventions need to focus on the cerebral cortex, providing patients with significant techniques to address their addiction. That this is a real addiction has been clearly shown in the autopsy-evaluated proliferation of nicotine receptor-binding sites in smokers’ prefrontal and temporal cerebral cortex’, Dr Pipe said.^3^

‘If you look at cigarette engineering, a science in itself, you will see the care that is taken to ensure rapid nicotine delivery. Essentially the cigarette is a sophisticated drug-delivery device.’ Dr Pipe pointed out.

‘As clinicians, we can significantly help our patients, by not haranguing them but adopting a constructive, unambiguous, simple and non-judgemental approach.We need to realise that we are dealing with a population of hardened smokers; smokers who have already tried and found it very difficult to stop smoking. In my view, all smokers who are trying to quit, except in the presence of very special circumstances, should receive pharmacotherapy for smoking cessation.’

There are three main therapeutic approaches: using nicotine replacement therapy (NRT), bupropion sustained release, or verenicline. ‘The chosen agent should be prescribed in appropriate doses and taken for as long as it takes to achieve cessation of smoking’, Dr Pipe advised.

A fundamental concept in NRT is to up-titrate according to the patient’s assessment of his level of comfort or distress. Caffeine levels may also rise on initiating smoking cessation therapy and this can be falsely interpreted as withdrawal symptoms – so patients need to be warned to lower their normal caffeine intake.

Bupropion is effective, with approximately 30% of smokers maintaining nonsmoking status at one year. It should be taken at a dosage of 150 mg twice a day but it does cause side effects of dry mouth, insomnia and shakiness.

The third-generation agent, verenicline, uniquely addresses the neurochemistry of tobacco addiction and has become the gold standard for smoking cessation therapies. It works as a partial agonist at the nicotine receptor site, alpha-4β2 nicotine acetylecholine receptor (nAChR), stimulating the release of sufficient dopamine to reduce cravings and withdrawal while simultaneously acting as a partial antagonist by blocking the reinforcing effects of nicotine.

Varenicline has become the gold standard in pharmacotherapy for smoking cessation because it achieves a 54% smoking cessation success rate, measured as non-smoking for a one-year period.^3^ Varenicline was synthesised by biochemists, based on cytisine, a natural plant alkoloid obtained from a plant growing in eastern Asia, known as Golden Rain, which was used by local people as a tobacco replacement.

Varenicline is safe and effective; particularly as continuing to smoke poses such high health risks. It does however cause some nausea and insomnia as it stimulates nicotine receptors in the gut. It can be used in combination with bupropion SR, as their different mechanisms can be helpful. In a study of the combination therapy,^4^ high smoking abstinence was achieved and no depressive symptoms were observed.

In conclusion, Dr Pipe urged clinicians to show clinical leadership and use emerging therapies for in-hospital smoking cessation approaches and in their daily practices.
